# The ethical cost of doing nothing

**DOI:** 10.1093/nsr/nwaa095

**Published:** 2020-06-04

**Authors:** Andrew J Parker

**Affiliations:** University of Oxford, Department of Physiology, Anatomy and Genetics, Oxford, UK

Concern has been raised about the ethics and risks of performing genetic interventions in macaque monkeys to study models of human neuropsychiatric conditions. Here I point out that, when the outcome of some situations is truly uncertain, human decision makers tend to evaluate these situations inconsistently. The consequences of this inconsistency are sometimes profound, as they lead policy makers and reviewers to ignore or discount the ethical costs of doing nothing. I conclude that there are fundamental problems with the ‘precautionary principle’ that Western policy makers often adopt to justify their decisions.

This article is written in response to a call for papers concerning the use of non-human primates for research purposes, particularly occasioned by the publication of papers from Chinese laboratories that performed gene-editing experiments in old-world monkeys (specifically Rhesus and Cynomolgus macaque monkeys) [1–3]. Whilst gene-editing and cloning experiments have been conducted for a number of years on mammalian species (mice and sheep), the combination of the use of non-human primates and the investigation of genes that create susceptibility to neuropsychiatric disorders led to an unusual degree of public commentary. Interestingly, a similar programme announced by Japan in 2014 did not create the same level of public concern, even though it uses gene-editing procedures in new-world monkeys (marmosets) with the explicit aim of being ‘a means to eventual better diagnoses and treatments of human brain disorders’ [4].

I begin with three observations about the specific research work that has prompted a call for this set of discussion papers. First, one can ask whether gene manipulation that is undertaken in a monkey is inherently more dangerous than other types of gene manipulation. The answer to this is that it is probably not more dangerous. One assessment of the risk with monkeys might be to compare this with the risks associated with gene manipulation of pathogenic agents, such as bacteria and viruses. Here, manipulation has been carried out to understand how the organisms are pathogenic to humans or animals and what could be done to alleviate this. Nonetheless, a pessimistic view of risk aversiveness would argue that there is always a risk that pathogenic qualities could be enhanced by such manipulation. Clearly, careful controls are undertaken to ensure that superpathogens are not released. If anything, the controls that can be applied to these experimental model animals, which have been genetically manipulated, are easier to implement than controls required to guard against inadvertent transmission of altered bacteria or viruses.

The second question that is specific to the research work on gene manipulation in monkeys is whether the normal controls over ethical treatment of the animals are somehow inadequate to cover this kind of scientific study. There clearly are questions about the degree of harm or suffering that might be caused to the animals by specific genetic interventions. However, these do not materially differ from the animal's perspective, simply because the intervention was genetic rather than environmental. It might separately be argued that because this manipulation is so new we have very little experience or knowledge than can be applied to understand what might be the consequences of a specific genetic intervention. We do however have considerable experience from selective breeding in domesticated animals of the consequences of selection in favour of or against certain characteristics. For example, some fighting dogs have been bred selectively over generations to increase the level of aggressiveness of the strain of animals.

Third, for one of the scientific studies [5] a gene-component specific to humans was introduced into the monkey genome. Introduction of human genes into mammals is not new. Indeed, it is the basis of much current, immunological research with mice, which can be ‘humanized’ to create a model animal that reproduces many of the characteristics of the human immune system in a mouse's body [6].

What characterizes these concerns is a claim that the scientists (and the ethical review process that the studies went through before they were conducted) underestimate the risk associated with the research work. It is contended that the true risk is systematically downplayed and it is further contended that the scientists’ estimation of the risk must be biased, so that the risk must be independently assessed by persons not involved in the work. Conceptually, a risk assessment is independent of an ethical assessment, but within current scientific review procedures, the two issues are often dealt with together.

In Western mythology and philosophy, the worst-case scenario is often referred back to the story of Pandora. In origin, the story is Greek but has been retold many times over. Pandora carries a vessel or box into which the gods have captured and contained evil. When the box is opened, the evil escapes, the Golden Age of a perfect humanity comes to an end and the state of the world is changed forever. As a mythological explanation of why the world is imperfect, it is a powerful image that persists even today. Some of the commentary on the recent Chinese experiments promotes the same vision of an irreversible and categorical change [7].

The need to have a clear appraisal of the value or otherwise of gene intervention in monkeys is clear. But it is also apparent that when making such appraisals, humans include elements of thinking that are in truth irrelevant to the scientific evaluation, but are nonetheless associated with value-judgments made by different human commentators. It is beyond the scope of a brief commentary to explore all the different dimensions of these additional elements, so the core of this article focusses on just one. This is the weakness of human decision making in circumstances where there is a great deal of uncertainty.

Risk aversion is a well-known characteristic of decision-making by humans. Under circumstances where the costs of choosing wrongly are potentially high and information about the likelihood of different outcomes is poor, there are good reasons for avoiding decisions that lead to risky outcomes. An inability to avoid risk is often a sign of poor mental state. In the extreme, behaviour that is deliberately risk-seeking can be truly pathological, as for example with gambling addictions. Within ethical debates, the desire for risk aversion is often elevated to the status of a principle, namely the so-called ‘precautionary principle’.

The basic impact of risk aversion on rational decision-making is illustrated nicely by the Ellsberg paradox (named after Daniel Ellsberg [8] and featured recently by Tim Harford in the *Financial Times* [5]). A person is offered a random draw from two boxes, each containing 100 balls. The first box contains exactly 50 red and 50 black balls. The second box also has 100 balls but an unknown mixture of red and black. The person is told that drawing a red ball will win them $100 and drawing a black ball will win nothing. The person is informed about the distributions of balls in each box and offered the choice of drawing from either box. Although there is no specific reason or incentive to do so, most people avoid the uncertainty associated with drawing from the second box and choose to draw from the first box.

The paradox really emerges when the ball chosen from the first box has been returned to its original place and people are offered a second chance to draw. However, the second draw is on the basis that the black ball wins $100. Now, although it might seem initially that something has changed on the basis of seeing the colour of the first ball drawn, there is actually no gain of information at all. The first ball has been drawn from a box for which the actual probability distribution has already been specified at 50:50. There is therefore no updating of information arising from the first draw.

Therefore, purely in terms of prior probabilities, if it was right to avoid the second box when hoping for a red ball, as happened on the first pick, then it must be also right to choose in favour of the second box when hoping for a black ball. Nothing about the draw from the first box reveals any new information about the probability distribution of balls in the second box. Thus, if the person chooses a 50:50 chance of a red ball as the better option on the first draw, in comparison with their lack of knowledge about the probability distribution in the second box, then on the subsequent draw people ought to choose from the second box. The first box cannot be favourable on both occasions, that is for drawing red on the first draw and black on the second draw. For, if the first box is better on the first draw in trying to achieve red, a rational agent must be assuming that there are fewer red balls than black in the second box. Nothing has changed for the second draw and therefore the rational agent ought to choose the second box, when black is the favoured outcome. Nonetheless people tend to avoid the second box and still choose the first at 50:50 on the second draw. Uncertainty seems to be something that promotes a failure of rational calculation in people.

We know from a lot of other experimental findings in decision-making that people are able to behave rationally when the priors are clear and well understood. But this rational behaviour is challenged when there is uncertainty about what the priors might be. In the Ellsberg case, the uncertainty ought not to change the choice behaviour but nonetheless does impact on the actual choices made. A number of reasons can be advanced about why this might be so. It could be that people mistrust the statements made by the experimenters. It could be that people attempt to bring information from other similar situations into the evaluation of the Ellsberg case. Whatever the reasons, the participants clearly dislike uncertainty to an extent that is difficult to find rational.

It is this mis-thinking that permeates the precautionary principle, as applied to the evaluation of research proposals. In evaluating how pursuit of a new research proposal would change things, we are inevitably being asked to perform the equivalent of picking from the second box, the one with the unknown distribution of outcomes. In sticking with the present state of affairs and doing nothing, we are effectively choosing from the first box: we know that things might improve by themselves or they might get worse, but we have a better estimate of the likelihood of each of these possibilities. The research route appears to be riskier because it adds uncertainty to each of the possible outcomes, so there is a hesitancy about adopting it.

What this issue really highlights is that there is a distinction to be made between uncertainty and risk. This distinction has been made in models of economic behaviour. Risk indicates that the outcome is uncertain but the parameters of that uncertainty can be usefully estimated. Once the parameters of the uncertainty are available, a rational choice can be made to place some resources at risk with the prospect of a likely gain. Under true uncertainty, the risk cannot be calculated. Unfortunately, under many circumstances, humans make decisions about uncertain situations as if they were inherently high risk. Humans also behave differently towards true uncertainty in comparison with high risk, in that they do not even order their preferences consistently when the prospect of gain is changed from red to black within the Ellsberg paradigm.

The implications of this general conclusion for formulation of public policy are considerable. I am concerned here primarily with the review process for studies in biomedical sciences that require both ethical consideration and risk assessment. Moving into the realm of real-world decisions, a review panel would be faced with a decision about whether to approve a specific research proposal. The considerations that apply to risk are different from those that should apply to true uncertainty. For risk, the possible outcomes need to be considered and ways need to be found to reduce risk. Ultimately, if the identifiable risks are too high, the research proposal might be declined. That would be a rational, precautionary approach, as the known risk can be avoided ultimately by not performing the study. For the case of true uncertainty, the issues are different. What we really want to reduce in this case is the uncertainty and one thing that is 100% clear is that abandoning the research study leaves everybody as uncertain as they were beforehand. Thus, treating true uncertainty as if it was just the same as risk will lead to incorrect decisions.

In the real world of research proposals in biomedical science, there is a further point. In our discussion of the Ellsberg paradox, there was no penalty for the wrong choice. That is to say, the consequences of drawing the wrong colour of ball out of the box are zero. In the real world of biomedical science, there are multiple prices potentially payable for making the wrong decision. There are many direct costs, such as the loss of research resources. There are also real potential harms, such as using animals to conduct experiments that are ultimately inconclusive. These need to be recognized and, where possible, assessed.

However, there are also indirect costs that are being continuously incurred and harms that are being continuously endured. These costs and harms are primarily incurred by other humans: that is, those who are not involved in the work. First, there are individuals who suffer as a consequence of some of the genetic conditions that are being investigated in the non-human primate work. Gaining a proper non-human primate model of these disease conditions is an important step to alleviation of these human conditions. One should not fall into the mistake of assuming that the only use of such a model is to build a case for genetic intervention in humans. These model systems are also important for providing a test-framework in which experimental treatments could be attempted. Second, there is a wider burden on human families and society as a whole that arises from the need to support individuals who carry the genetic conditions. Both of these are penalties that arise from choosing not to do anything. In other words, these outcomes bring an ethical cost of doing nothing.

The critical articles have raised concern about the feelings of a macaque monkey that carries one of the human specific genes with neuropsychiatric consequences. These articles argue that this concern is sufficient to stop further research investigation. Animal suffering is an important consideration and we should not recklessly bring this about. But, in respect of these experimental studies, there also would have to be an ethical concern about not doing things when we could do them. If it were true that genetic intervention brings about suffering in animals that cannot be alleviated in any acceptable way, then it is equally true that allowing natural human reproduction to continue creating human babies that will endure life-long suffering also brings an ethical cost. Application of the precautionary principle (along the lines of ‘first, do no harm’) appears to be inadequate to deal with circumstances where a constant stream of harm is already being created.

There is a cultural, religious strand in Western Christian thinking that has argued that suffering is in itself a path to more virtuous behaviour. It is unclear whether this is an additional consideration that factors into thinking that some human suffering is acceptable. This is not to say that these decisions are easy. However, in relation to the research under discussion here, it is essential to be clear that ‘just stopping the research’ is not a neutral option. Not doing things also has ethical consequences.
